# SLAM-SAP-Fyn: Old Players with New Roles in iNKT Cell Development and Function

**DOI:** 10.3390/ijms20194797

**Published:** 2019-09-27

**Authors:** Devika Bahal, Tanwir Hashem, Kim E. Nichols, Rupali Das

**Affiliations:** 1 Comparative Medicine and Integrative Biology Program, College of Veterinary Medicine, Michigan State University, East Lansing, MI 48824, USA; bahaldev@msu.edu; 2 Department of Physiology, College of Natural Science, Michigan State University, East Lansing, MI 48824, USA; hashemta@msu.edu; 3 Department of Oncology, St. Jude Children’s Research Hospital, Memphis, TN 38105, USA; kim.nichols@stjude.org; 4 Department of Physiology, College of Human Medicine, Michigan State University, East Lansing, MI 48824, USA

**Keywords:** natural killer T cells, signaling lymphocyte activation molecule (SLAM), SLAM-associated protein, Fyn, lineage development, cell fate, transcription factors, cytokine, antitumor response

## Abstract

Invariant natural killer T (iNKT) cells are a unique T cell lineage that develop in the thymus and emerge with a memory-like phenotype. Accordingly, following antigenic stimulation, they can rapidly produce copious amounts of Th1 and Th2 cytokines and mediate activation of several immune cells. Thus, it is not surprising that iNKT cells play diverse roles in a broad range of diseases. Given their pivotal roles in host immunity, it is crucial that we understand the mechanisms that govern iNKT cell development and effector functions. Over the last two decades, several studies have contributed to the current knowledge of iNKT cell biology and activity. Collectively, these studies reveal that the thymic development of iNKT cells, their lineage expansion, and functional properties are tightly regulated by a complex network of transcription factors and signaling molecules. While prior studies have clearly established the importance of the SLAM-SAP-Fyn signaling axis in iNKT cell ontogenesis, recent studies provide exciting mechanistic insights into the role of this signaling cascade in iNKT cell development, lineage fate decisions, and functions. Here we summarize the previous literature and discuss the more recent studies that guide our understanding of iNKT cell development and functional responses.

## 1. Introduction

Conventional T cells (T_con_) develop in the thymus and are characterized by the presence of a diverse spectrum of T cell antigen receptors (TCRs) that recognize a wide array of peptide antigens. T_con_ recognize these antigens when presented by highly polymorphic cell-surface molecules, namely, the major histocompatibility complex I (MHC I) or MHC class II proteins. However, there exist several subsets of T lymphocytes that develop in the thymus but do not recognize peptide antigens, have limited TCR expression, and are relatively rare in number when compared to T_con_. Despite their lower incidence, most of these “unconventional” T cells are fast responders to antigenic stimulation and play pivotal roles in host immune responses. Some of these atypical T cells include but are not limited to CD1-restricted natural killer T (NKT), MR1-restricted mucosal associated invariant T (MAIT), and γδ T cells. NKT cells are evolutionarily conserved innate-like T lymphocytes that recognize lipid antigens and can be further classified based on their TCR expression: Invariant or type I NKT cells, and diverse or type II NKT cells [1]. Our overall knowledge of type II NKT cells remains limited due to the paucity of reliable reagents to study these cells. In this article, we review our current knowledge of invariant NKT cells focusing on the role of SLAM-SAP-Fyn signals in their development and function.

## 2. Invariant Natural Killer T Cells

Type I NKT cells, also known as the “invariant” NKT (iNKT) cells express a semi-invariant Vα14-Jα18 TCR that pairs with a limited number of β-chains (Vβ8.2, Vβ7 or Vβ2) in mice [2,3]. Human iNKT cells also express a restricted TCR that is made up of the Vα24-Jα18 and Vβ11 TCR chains [2,3]. Both human and murine iNKT cells recognize glycolipid antigens, such as α-galactosylceramide (α-GC), when presented by MHC class I-like molecule, CD1d [4,5,6]. Of the various antigens that are known to activate iNKT cells, α-GC, which was originally isolated from the sea sponge *Agelas mauritianus* [6] has been most extensively characterized. Indeed, the development and use of α-GC-loaded CD1d tetramers provide the most reliable detection of iNKT cells and have greatly contributed to our knowledge of iNKT cell development, tissue distribution, activation and function. 

In mice, iNKT cells are most abundantly found in the liver but are also present in the spleen and lungs where they represent 20–30% or 1–2% of all lymphocytes, respectively [7,8]. They are also found in the bone marrow, gut and skin [9,10] and to a lesser extent in the peripheral lymph nodes [11]. Human iNKT cells are most abundantly found in the liver (where they comprise of 1% of the total lymphocytes [7,8,12], but are rare (often less than 0.1% of T cells) and highly variable in peripheral blood [13]. Two main subsets of iNKT cells exist in mice: CD4^+^ and CD4^−^CD8^−^ double negative (DN), although the functional differences between these populations are unclear [14]. Specifically, no distinct differences in their ability to produce IFN-γ and interleukin (IL)-4 production has been observed [15]. Yet in another study [11], iNKT cells were found to be extremely diverse in their cytokine response. Other studies in mice have demonstrated that the CD4^+^ iNKT cells play an important role in the induction of CD8^+^ regulatory T cells [16] and also exhibit a suppressive role in the development of diabetes in non-obese diabetic mice [17]. With respect to their anti-tumor activities, it has been shown that murine DN (CD4^−^) iNKT cells, particularly from the liver, have higher lytic activity than their CD4^+^ counterparts [15]. Interestingly, differential production of the pro-inflammatory cytokine, IL-17 by CD4^−^ and CD4^+^ liver iNKT cells has been reported [11], which could explain the superior anti-tumor response of the CD4^−^ subset. Invariant NKT cells generally express several receptors that are characteristic of natural killer (NK) cells [18], including NK1.1, Ly49, and NKG2D. However, some murine NK1.1^−^ iNKT cells can be found in the thymus as well as the periphery [19,20,21]. These are likely the immature cells that have recently egressed from the thymus [19,20] or activated cells that have down regulated their NK1.1 expression [22]. 

In humans, iNKT cells are CD4^+^, DN, or CD8^+^ [23,24,25] with distinct cytokine profiles i.e., CD4^+^ iNKT cells are the exclusive producers of Th2 cytokines like IL-4 and IL-13 whereas DN iNKT cells have a strict Th1 profile [26]. However, studies have also shown that both CD4^+^ and the CD4^−^ iNKT cells can secrete high amounts of IFN-γ, GM-CSF and TNF-α with varying levels of IL-2, IL-4, and IL-5 [27,28]. In most cases, CD4^+^ iNKT cells produce more IL-4 and other Th2 cytokines than the CD4^−^ iNKT cells [24,28,29]. Consistent with this observation, IL-4 and IL-13 producing CD4^+^ iNKT cells are found in the lungs of chronic asthma patients [30]. Human iNKT cells also express some markers of the NK linage including NKG2D, CD94, and NKG2A that are mainly restricted to CD4^−^ iNKT cells [31]. Accordingly, CD4^−^ iNKT cells mount direct cytotoxicity against tumor target cells which can be mediated via TCR-CD1d interactions [32,33] or directly via NKG2D engagement [31].

## 3. Development of iNKT Cells

The distinctive phenotypic and functional attributes of iNKT cells are accredited to their unique developmental program that is tightly regulated by several transcription factors and signaling molecules [1,34,35]. Invariant NKT cells undergo positive or negative selection so that the potentially autoreactive cells can be eliminated and functional cells with low affinity for self-antigens are retained [1,35].

## 4. Positive Selection

Development and positive selection of iNKT cells is critically dependent on CD1d expression, particularly on CD4^+^CD8^+^ double positive (DP) cortical thymocytes, as such iNKT cells are absent in *Cd1d***^−/−^** mice [36,37,38]. Additionally, for efficient iNKT cell selection, CD1d must recycle through specific intracellular pathways, where it can be loaded with endosome- or lysosome-derived antigens [3]. Although it remains to be formally established, it is believed that the ligand for positive selection of iNKT cells is most likely a self-antigen. Isoglobotrihexosylceramide (iGb3), a weak endogenous glycosphingolipid antigen with structural similarities with α-GC was thought to be a potential ligand for positive selection [39,40,41,42]. However, evidence regarding the role of iGb3 in iNKT cell development has been conflicting [43,44,45]. Several other molecules including β-linked monoglycosylceramides, such as β-glucosylceramides (β-GluCer) have also been proposed to be endogenous ligands for iNKT cells, and synthetic preparations of C12:0 and C24:1 β-GluCer have been shown to be strong iNKT cell agonists [46,47,48]. However, recent studies by Kain et al. demonstrate that β-GluCer has no iNKT cell stimulatory properties and suggeste that contamination of synthetic β-linked glycolipids with α-anomers could have contributed to iNKT cell activity in prior studies [49]. Using multiple sophisticated immunological and enzymatic approaches, Kain et al. [49] provide strong evidence that endogenous selecting ligands for iNKT cells are α-linked monoglycosylceramides, including α-galactosylceramide and α-glucosylceramide and not iGB3. Additionally, they also provide evidence that supports the idea that the endogenous α-glycosylceramides levels are regulated by a two-step catabolic process rather than rapid de novo synthesis of the ligand. Collectively, these studies provide novel insights that help discern some of the unexplained prior observations related to iNKT cell biology, such as the presence of iNKT cells in β-D-GalCer synthase-deficient mice [50], differential reactivity of thymic iNKT cells to α-GluCer and α-GalCer CD1d tetramers and their bias to Vβ7 and Vβ8 iNKT cells, respectively [51], and the basis for tissue specific recognition of CD1 molecule by the iNKT cells [52]. 

As mentioned earlier, iNKT cells have a limited Vβ usage which could either be due to pairing preferences with the invariant TCR α-chain and/or a consequence of positive selection. The possibility of pairing bias is ruled out as transgenic expression of the invariant TCR α-chain (Vα14-Jα18) in *Cd1d***^−/−^** mice exhibit a diverse Vβ repertoire [40]. Interestingly, only the cells expressing the Vβ7, Vβ8 and Vβ2 respond to endogenous ligands and iGb3, with an affinity hierarchy (Vβ7 > Vβ8 > Vβ2) [40] that directly correlates with the limited Vβ expression found naturally in iNKT cells. Based on these observations [40] and additional studies [5,53], it is now believed that iNKT cells acquire their restricted TCR β-chains following positive selection.

## 5. Negative Selection

When engaged by high-avidity antigen or abundant self-antigen, iNKT cells are likely to undergo negative selection. Indeed, studies demonstrate that addition of α-GC (strong agonist) to a fetal thymic organ culture (FTOC) induces a dose-dependent disappearance of iNKT cells [54]. In contrast, the delayed introduction of α-GC in vitro or in vivo, after the development of iNKT cells, does not deplete these cells [54]. Moreover, overexpression of CD1d on dendritic cells abrogates iNKT cell development [55] and the residual cells exhibit increased Vβ2 TCR usage that respond poorly to antigenic stimulation [55]. It is believed that negative selection occurs during early stages of development, probably at the same time as positive selection. To that end, studies have shown that the transcription factor, Nur77 (a molecule associated with negative selection) is expressed by very immature iNKT cells [56]. 

## 6. Maturation and Functional Differentiation of iNKT Cells

**Figure 1:**
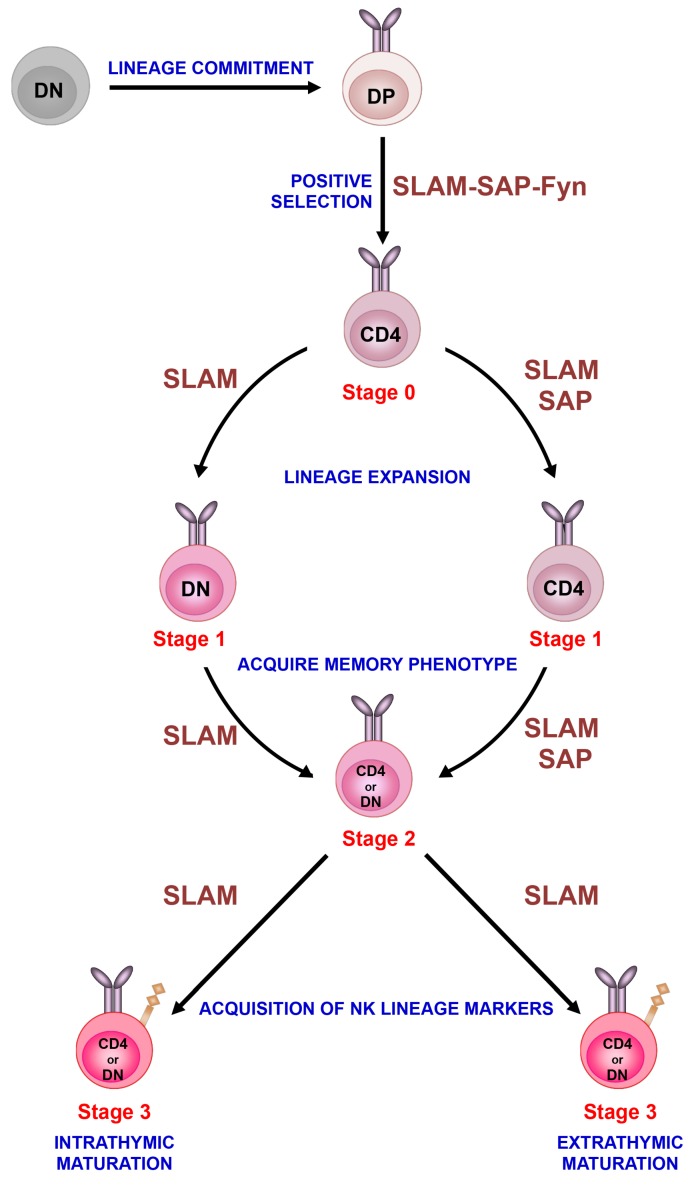
Sequential lineage development model. Invariant NKT cells develop in the thymus following homotypic interactions between CD4^+^CD8^+^ DP thymocytes that express the invariant TCR. After positive selection, immature iNKT cells progress through distinct stages of maturation that are characterized by differences in the surface expression of CD24, CD44 and NK1.1. Immature iNKT cells (stage 0) undergo lineage expansion (stage 1) and acquire a memory phenotype (stage 2) as well as markers of the NK cell (NK1.1) lineage (stage 3). Although most immature iNKT cells emigrate from the thymus at stage 2 and acquire NK1.1 expression in the periphery, a subset of stage 2 cells acquires this marker in the thymus and remains as long-term residents. The development of iNKT cells is critically regulated by the signaling lymphocyte activation molecule (SLAM) family of receptors (referred to as SLAM), SAP and Fyn. Based on recent studies, we have placed these signaling molecules along the iNKT cell developmental program at the various stages where their activity appears to be critical for iNKT cell ontogeny.

**Figure 2:**
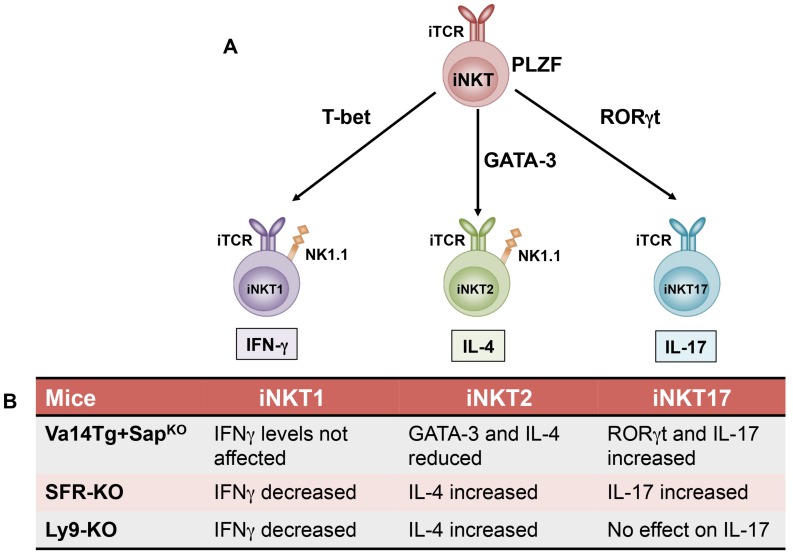
Invariant NKT cell lineage diversification model. (**A**) As iNKT cells develop in the thymus, they acquire their functional characteristics that is critically dependent on the transcription factor PLZF. Invariant NKT cells can be further classified into functionally distinct subsets such as iNKT1, iNKT2, and iNKT17 based on their cytokine production profile and respective expression of signature transcription factors such as T-bet, GATA-3, and RORγt. (**B**) Recent studies highlight a role for SLAM family of receptors (SFR KO: Lack all the 7 receptors), specifically Ly9 and SAP in the regulation of iNKT cell fate decisions and are summarized in the table. iTCR: Invariant T cell receptor.

Following positive selection, iNKT cells go through distinct stages of maturation that are marked by the sequential acquisition of surface markers such as CD24, CD44 and NK1.1 [35] (sequential lineage developmental model; Figure 1) and/or expression of key transcriptional factors [57] (lineage diversification model; Figure 2). According to the conventional classification (sequential lineage developmental model), the most immature NKT cells are defined as CD24^hi^CD44^lo^NK1.1^−^ (stage 0), followed by CD24^lo^CD44^lo^NK1.1^−^ (stage 1), CD24^lo^CD44^hi^NK1.1^−^ (stage 2), and CD24^lo^CD44^hi^NK1.1^+^ (stage 3). At stage 0, iNKT cells are present in very low numbers in the thymus. As they progress to stage 1, there is a burst of proliferation and the iNKT cells either remain CD4^+^ or become double negative (DN) by down regulating the CD4 surface expression. As these cells progress to stage 2, they upregulate CD44 and acquire a memory phenotype [35]. Most of the CD24^lo^CD44^hi^NK1.1^−^ cells exit the thymus at this stage and home to peripheral tissues, where they rapidly express markers of activation (CD122, CD69) as well as NK1.1, NKG2D and Ly49 [58]. However, NK1.1 is not expressed in all mouse strains and does not correspond to functional capacity of iNKT cells. Thus, recent studies have used expression of specific transcription factors to classify iNKT cell subsets (lineage diversification model; Figure 2A) since these patterns correlate directly to functional maturation [57]. Accordingly, iNKT cells are classified as NKT1, NKT2, and NKT17, based on their expression of the transcription factors T-bet, GATA-3 and RORγt and their respective cytokine (IFN-γ, IL-4 and IL-17) production profiles [57,59] (Figure 2A). 

These iNKT cell subsets (NKT1, NKT2, and NKT17) arise from a common progenitor cell that has high expression of the transcription factor promyelocytic leukemia zinc finger (PLZF), the key regulator of iNKT cell development and function [60,61]. In the absence of PLZF, there is a dramatic reduction in iNKT cell numbers in the thymus and importantly PLZF-deficient cells do not acquire the characteristic iNKT cell activated phenotype [60,61]. Prior studies have shown that T-bet is critical for the final stages of iNKT cell maturation [58,62]. In the absence of T-bet, reduced iNKT cell numbers are found at stage 2 (CD44^hi^NK1.1^−^) [62]. Furthermore, T-bet-deficient iNKT cells exhibit defective IFN-γ production and reduced cytolytic activity [58,62,63]. The transcription factor GATA-3 regulates the generation of the CD4^+^ iNKT cells (NKT2) at stage 1 [64]. GATA-3 deficient iNKT cells fail to produce activation-induced Th1 and Th2 cytokines [65,66]. Finally, the transcription factor RORγt not only facilitates iNKT cell development at the DP stage [67,68], but is also required for the differentiation of a unique subpopulation of iNKT cells (NK1.1^−^ CD4^−^) that is capable of producing IL-17 in response to TCR stimulation [11]. Thus, the lineage diversification model more or less correlates with the conventional classification as NKT1 cells are predominantly stage 3 and NKT2 cells are stages 1 and 2 [57]. While NKT1 and NKT2 are found primarily in the liver and spleen, NKT17 is found in the lymph-nodes and are far fewer than the other iNKT subsets [59].

Several other transcription factors such as NFκB, c-Myc, c-Myb, Runx1, Egr2, and HEB regulate iNKT cell development as ablation of any of these genes results in a severe impairment in iNKT cell development [34,59]. While many of these transcription factors also regulate T_con_ development, the signaling pathways that govern iNKT cell development are more distinct. Unlike T_con_, development of iNKT cells is critically dependent on the SLAM (signaling lymphocytic activation molecule) receptors, the *Sh2d1a*-encoded adaptor molecule SAP (SLAM-associated protein) and the Src- kinase Fyn. In the section below, we discuss prior studies and highlight the more recent reports that provide new insights into the role of SLAM-SAP-Fyn signaling in iNKT cell development, lineage differentiation and function. 

## 7. SLAM-SAP-Fyn Axis in iNKT Cell Development

Fyn was the first signaling molecule that was shown to be selectively important for iNKT cell development. While ontogeny of T_con_ is normal in the absence of Fyn, iNKT cell development is nearly ablated in *Fyn*^−/−^ mice [69,70]. Fyn binds to SAP, which is highly expressed in T_con_ and NK cells and is essential for their function [71]. However, *Sap*^−/−^ mice have no obvious defects in the number or maturation of T_con_ or NK cells [72]. In sharp contrast, we and others have showed that *Sap*^−/−^ mice and patients with the immunodeficiency X-linked lymphoproliferative disease (XLP), that lack or express germline mutations in the *SH2D1A* gene, have almost no iNKT cells [73,74,75]. Collectively, these studies established a critical and linage-specific role for SAP in the development of iNKT cells. The developmental defects observed in *Sap*^−/−^ mice are similar to *Cd1d*^−/−^ animals, however, the reduced iNKT cell numbers in *Sap*^−/−^ mice is not due to impaired CD1d expression or function [73]. Furthermore, overexpression of pro-survival molecules Bcl-2 and Bcl-xL or constitutive activation of NF-κB failed to restore iNKT cell numbers in *Sap*^−/−^ mice [76] suggesting that SAP’s role in iNKT cell development extends beyond the provision of survival signals.

Although SAP and Fyn are both required for iNKT cell development, the phenotype of *Fyn*^−/−^ mice is less severe in comparison to *Sap*^−/−^ animals, indicating potential compensation by other Src kinases such as Lck. Consistent with this notion, iNKT cell numbers are reduced in *Lck*^−/−^ mice [69,70]. The SH2 domain of SAP binds directly to the SH3 domain of Fyn and this interaction is required during iNKT cell ontogeny [77]. Employing cells or mice that either expressed the wild-type or a mutant version of SAP that cannot bind to Fyn (*Sap*^R78A^), we demonstrated that the SAP-Fyn interaction is critical for efficient iNKT cell development in vitro as well as in vivo [77]. Strikingly, the defect in iNKT cell development induced by the *Sap*^R78A^ mutation is not as severe as that observed in mice lacking the expression of SAP itself [77], suggesting that in iNKT cell progenitors, SAP may signal independent of Fyn binding. In support of this notion, in vitro studies demonstrate that SAP can also bind to Lck and this interaction is not dependent on the R78 residue [78].

The developmental stages at which SAP and Fyn are required remain unclear. In a prior study transgenic expression of the invariant TCRα chain Vα14-Jα18 completely restored CD1d-restricted NKT cell numbers in *Fyn*^−/−^ mice, indicating that Fyn is required early in NKT cell development, prior to TCR rearrangement [79]. However, contrary to this, a later study [80] showed the presence of CD24^high^tetramer^high^CD69^high^ NKT cells in *Sap*^−/−^ and *Fyn*^−/−^ mice, suggesting that in the absence of SAP or Fyn, iNKT cell development is halted after generation of the TCRα chain, during or immediately after positive selection.

The membrane proximal mediators that recruit SAP-Fyn during iNKT cell development belong to the SLAM family of immunoreceptors. The SLAM family of receptors (SFRs) consists of seven members [81,82] including SLAMF1 (SLAM/CD150), SLAMF2 (CD48), SLAMF3 (Ly9/CD229), SLAMF4 (2B4/CD244), SLAMF5 (CD84), SLAMF6 (Ly108/CD352), and SLAMF7 (CRACC/CD319). Except for 2B4 and CD48 (which bind to each other), the SFRs are homotypic self-associating receptors expressed by cells of hemopoietic origin [71,83]. SFRs have two to four unique immunoreceptor tyrosine-based switch motifs in their cytoplasmic domains to which SAP can bind [83]. In T_con_, SFR signaling is mediated in part by SAP via Fyn, which in turn phosphorylates the SLAM receptors. The SFRs then provide docking sites for downstream signaling molecules [71,83]. However, in the absence of SAP, SFRs can also signal through other SH2 -domain containing molecules such as the lipid phosphatase SHIP-1 or the protein phosphatases SHP1 and/or SHP2 [84,85].

Analysis of surface expression of the SFRs on developing thymocytes revealed that 2B4 is not expressed on mouse thymocytes [80,86], while Ly9 and CD84 have weak expression on DP cells [80]. In contrast, SLAM and Ly108 have the highest expression on DP cortical thymocytes [80,86]. While SLAM is rapidly downregulated after the DP stage, Ly108 expression persists until stage 1 (CD24^lo^) [80]. However, *Slamf1***^−/−^** or *Slamf6***^−/−^** have no defect in iNKT cell development [80], consistent with prior studies [87,88,89]. Subsequent studies using *Slamf1***^−/−^** and *Slamf6***^−/−^** bone marrow chimeras in lethally irradiated *Jα18***^−/−^** mice revealed that the developmental defect is at the transition from CD24^hi^ (stage 0) to CD24^lo^ (stage 1), suggesting that both SLAM and Ly108 are required for iNKT cell development after positive selection [80]. This has now been confirmed by several recent studies [86,90,91,92]. However, the iNKT cell developmental defect is more profound in the absence of Ly108 than in the absence of SLAM [80]. Consistent with this observation, overexpression of Ly108 but not SLAM in SFR-KO mice (which lack all the seven SFR members) is sufficient to restore the severe defect in iNKT cell ontogeny [93]. Furthermore, studies in the SFR-KO mice suggest that multiple SAP-associated SFRs, besides SLAM and Ly108, are required for iNKT cell development which include Ly9 and 2B4/CD48 but not CD84 and CRACC [86]. Yet other studies have shown that SAP-associated Ly9 [94] and SAP-independent SFRs CRACC and the less-known SLAMF8 [95] play a negative role in iNKT cell development. This is evident by increased numbers of thymic PLZF^hi^ iNKT cells in mice lacking these specific SFRs [94,95]. Collectively, these studies suggest that differential SFR expression can positively or negatively influence iNKT cell development. Importantly, in the absence of SAP, the positive activating receptors can also transmit inhibitory signals to negatively regulate iNKT cell development in *Sap***^−/−^** mice [86,92]. However, SFR-associated inhibitory signals play a small part in iNKT cell development which is SHIP-1 independent [86].

Mechanistically, SFRs contribute to iNKT cell development after positive selection by recalibrating the TCR signal strength and promoting cell survival via Bcl-2 expression [93]. In the absence of SFRs, iNKT cells proliferate rapidly but fail to survive and exhibit a high expression of Nur77 and CD5, indicating increased TCR signal strength [93]. Furthermore, SFR-KO iNKT cells have a skewed TCR Vβ repertoire and upregulate inhibitory receptors such as PD-1, Lag-3 and CD160 [93]. Previous studies have shown that following TCR stimulation, *Sap*^−/−^ or *Fyn*^−/−^ T_con_ cannot recruit signaling intermediates PKC-θ and Bcl10 to the immunological synapse and also have reduced NF-κB nuclear translocation [96]. Furthermore, mice deficient in PKC-θ or Bcl10 have severely reduced iNKT cell numbers in the thymus and spleen indirectly linking the SLAM-SAP–Fyn complex to TCR-induced signals [97,98]. Consistent with this notion, recent studies by Chen et al. demonstrated that SFRs promote iNKT cell development in part by activating the CARMA1–Bcl10–Malt1 (CBM) complex [86]. They further demonstrated that CARD9 (caspase recruitment domain family of adaptor member 9)-containing CBM complex is partially required for iNKT cell development in the thymus but not for their peripheral maintenance [86].

## 8. SLAM-SAP Signals in iNKT Cell Lineage Differentiation

The signaling mechanisms that drive thymic fate decisions of iNKT cells are not fully understood. Recently, Lu et al. demonstrated that SFRs are not essential for the development of CD24^hi^ iNKT cells (stage 0) but are increasingly required as the cells progress through the various stages of development, the highest dependence on SFRs being by the most mature iNKT cells (stage 3) [93]. Furthermore, SFR-KO mice display reduced incidence of T-bet^+^PLZF^lo^ iNKT1 cells but increased proportions of GATA-3^+^PLZF^hi^ iNKT2 and RORγt^+^PLZF^int^ iNKT17 cells, highlighting a novel role of SFRs in iNKT cell lineage differentiation [93]. In agreement to this, *Ly9*^−/−^ mice have increased thymic iNKT2 but very few iNKT1 cells [99]. However, this skewed subset distribution is not observed in the periphery [99], pointing to the possibility that the sub-lineage committed iNKT cells retain some degree of plasticity. Indeed, once they exit the thymus, iNKT cells can reprogram themselves depending on the signaling cues they receive in the periphery [57].

Recent studies have also implicated SAP in the regulation of iNKT cell lineage expansion, though the mechanisms by which SFRs and SAP facilitate this process appear to be distinct (Figure 2). While SFRs regulate the expression of PLZF (but not Egr2) [86,93], SAP is critical for GATA-3 expression [100]. Moreover, unlike SFRs, SAP favors the generation of iNKT2 but not iNKT1 or iNKT17 cells [93,100]. SAP-deficient iNKT cells have significantly reduced levels of the iNKT2-associated transcription factors GATA-3 and PLZF, but abundantly express the transcription factor RORγt and are NK1.1^−^CD4^−^ [100]. Thus, it is likely that SAP regulates the generation of the CD4^+^ iNKT cell lineage and production of the Th2-type cytokines by modulating GATA-3 expression. Whether or not Fyn and/or SAP-Fyn interactions are important for iNKT cell fate decisions remain to be determined.

## 9. SLAM-SAP-Fyn in iNKT Cell Function

**Figure 3:**
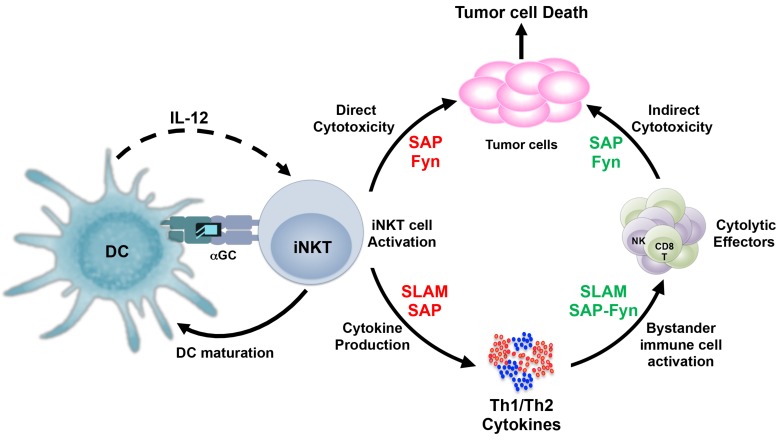
SLAM-SAP-Fyn regulate the antitumor mechanisms of iNKT cells. Invariant NKT cells recognize glycolipid antigens when presented by antigen presenting cells (such as DCs) and rapidly produce large amounts of Th1 and Th2 cytokines, which is dependent on SLAM family of receptors and may be SAP, in some cases. For direct cytotoxic response, iNKT cells critically rely on signals generated by SAP and the Fyn kinase, although their physical interaction with each other may not be necessary. Antigen-induced cytokine production by iNKT cells leads to activation of other immune cells, including CD8^+^T and NK cells. These cytotoxic effectors depend on the SLAM-SAP-Fyn axis for their cytokine production as well as cytolytic functions. Dependence on the SLAM-SAP-Fyn signals by iNKT cells and/or other immune cells (such as CD8^+^T and NK cells) are indicated by red and green fonts respectively.

Once activated via their invariant TCR, iNKT cells rapidly produce both Th1 and Th2 cytokines and up-regulate the expression of co-stimulatory molecules. Thus, iNKT cells can promote maturation of DCs and modulate the functions of NK, T and B cells [101,102,103]. Additionally, mature iNKT cells express cytolytic proteins (perforin and granzymes) [58,104,105] and can be induced to up-regulate death-promoting molecules (Fas ligand and TRAIL) [105,106,107]. These findings suggest that iNKT cells can mediate their anti-tumor activity via multiple mechanisms. Indeed, others and we have demonstrated that murine and human iNKTs mount potent cytotoxic responses to numerous CD1d+ tumors in vitro and in vivo [6,32,108,109,110,111]. However, the mechanisms that control iNKT cell anti-tumor functions are poorly understood. In this section, we discuss recent studies that provide new information on how SLAM-SAP-Fyn signaling regulates iNKT cell cytokine production and direct cytotoxic responses (Figure 3). SFR-KO mice produce significantly less cytokines (both IFN-γ and IL-4) following in vivo administration of α-GC [93]. This is not due to diminished iNKT cell numbers in these animals, as in vitro stimulation of SFR-KO splenic iNKT cells also exhibit reduced intracellular cytokine expression [93]. Consistently, SLAM-SLAM interactions between iNKT and dendritic cells are required for iNKT cell production of Th2 cytokines like IL-4 and IL-10 but not IFN-γ [112]. In contrast, activation of the Ly9 receptor with an agonistic monoclonal antibody impairs iNKT cell cytokine production [99]. Based on these reports, it appears that signals generated by SFRs can positively or negatively regulate iNKT cell cytokine production.

Given the critical role of SAP in iNKT cell development, studying its role in iNKT cell functions has been a predicament. We and others have demonstrated that expression of the Vα14-Jα18 transgene in *Sap*^−/−^ mice restores the generation of iNKT cells that bind to α-GC-CD1d tetramer and proliferate normally but fail to secrete cytokines in response to TCR ligation [76,100]. In our studies, Vα14Tg^+^*Sap*^−/−^ iNKT cells had an abnormal phenotype and produced significantly less IL-4 and IFN-γ [76]. Thus, it was difficult to distinguish whether the observed cytokine defects were because of SAP deficiency or due to the development of immature iNKT cells in the absence of SAP. In contrast, in a more recent study, the Vα14Tg^+^*Sap*^−/−^ iNKT cells were mostly mature (CD44^+^) and exhibited selective defect in IL-4 but not IFN-γ or IL-17 production [100]. The observed disparity in these studies may be due to the genetic background or differences in how the Vα14Tg mice were generated; however, both the studies suggest a role for SAP in iNKT cell cytokine production. Contradicting this idea, we [109] and others [113] have found that conditional deletion of SAP (SAP^−^: SAP-deficient and SAP^+^: SAP-sufficient) after iNKT cell development has no impact on antigen-induced cytokine production in vivo. Consistently, iNKT cell-dependent transactivation of other immune cells is comparable in SAP^+^ and SAP^−^ mice [109,113]. Collectively, these studies suggest that once iNKT cell development is completed, the deletion of SAP does not influence iNKT cell cytokine production. The reduced cytokine responses we previously observed in the Vα14Tg^+^*Sap***^−/−^** mice are likely due to SAP-deficiency during iNKT cell ontogenesis [76].

SAP-deficient T cells adhere normally to DCs but display poor adhesion to T and B cells [114,115]. Accordingly, we observe that purified SAP^−^ iNKT cells secrete cytokines normally when cultured with DCs but respond poorly when the antigen is presented by T (EL4) lymphoid cells [109]. Thus, it is not surprising that SAP^−^ mice exhibit normal in vivo cytokine production, given that these responses mostly depend on antigen presentation by DCs and not lymphoid cells. SAP relies on its association with Fyn to facilitate optimal Th2 cytokine production by activated CD4^+^ T cells [96,116]. However, the residual iNKT cells from *Fyn*^−/−^ mice [79] and those from *Sap*^R78A^ animals robustly produce IL-4 and IFN-γ [77], suggesting that neither Fyn nor its association with SAP is required for iNKT cell cytokine production.

Besides their immunomodulatory functions, iNKT cells can directly recognize and kill CD1d^+^ tumor cells [108,117]. By using highly purified SAP^+^ and SAP^−^ iNKT cells from the aforementioned conditional SAP knockout mice, we have demonstrated that SAP is critical for iNKT cell-mediated target cell lysis in vitro as well as for control of tumor growth in vivo [109]. Moreover, the suboptimal in vivo tumor clearance is not because of the inability of SAP^−^ iNKT cells to traffic to the tumor site but due to their reduced capacity to kill. This was further confirmed in DN3A4–1.2 NKT (1.2) cells stably transduced with lentiviruses encoding *Sap*-targeted shRNA sequences [109]. SAP-dependent defects in killing are not due to the altered expression of lytic molecules, death-promoting receptors or inhibitory proteins [109]. Rather, SAP^−^ iNKT cells form fewer conjugates with T-cell targets and exhibit reduced polarization of the microtubule-organizing center (MTOC) at the immunologic synapse (IS). Together, these studies accentuate a novel role for SAP in iNKT cell cytotoxicity and formation of a functional IS [109]. This dependence on SAP for cytotoxic response is not restricted to murine iNKT cells, as human iNKT cells transfected with *SAP*-specific but not control siRNAs fail to kill CD1d^+^ tumor cells [109].

SAP and Fyn as well as their physical interaction are necessary for NK cell cytolytic activity [72,118]. However, the rare iNKT cells obtained from *Fyn*^−/−^ and *Sap*^R78A^ mice have no defects in iNKT cell cytotoxicity [109]. Contrary to these observations, 1.2 cells stably expressing Fyn-shRNA sequences as well as *FYN*-silenced human iNKT cells both exhibit significant reduction in target cell killing indicating that Fyn is a critical mediator of iNKT cell lytic activity [109]. Thus, it is possible that *Fyn*^−/−^ or *Sap*^R78A^ iNKT cells do not reflect the functions of normal iNKT cells because of their altered thymic development that is independent of Fyn. Collectively, these studies emphasize that unlike in T_con_ and NK cells, SAP-Fyn interactions are critical for iNKT cell development, but these signals may be selectively required for specific iNKT cell functions.

Mutations in the *SH2D1A* gene resulting in X-linked lymphoproliferative disease (XLP) are associated with impaired iNKT cell development as well as defective immune cell activities, such as NK and CD8+ T cell cytotoxicity, T cell cytokine production, activation-induced cell death, and germinal center formation [81]; defects that are recapitulated in *Sap*^−/−^ mice [119,120,121]. The clinical manifestation of XLP is characterized by severe immune dysregulation following exposure to Epstein–Barr virus (EBV), including fulminant infectious mononucleosis (FIM), dysgammaglobulinaemia and lymphoma [81]. Although rare, XLP is often fatal with mean age of expression being less than 5 years and the mortality rate close to 100% by age 20. The only curative option for XLP is allogeneic hematopoietic stem cell (HSC) transplantation [122,123]. However, recent studies by Rivat et al. provide “proof of principal” that gene therapy is a possible treatment option for XLP [124]. In this study, *Sap*^−/−^ mice that received HSCs transduced with lentiviral vectors expressing wild type SAP had significantly high NKT cell numbers and increased NK-cell cytotoxicity. Furthermore, these animals exhibited increased basal immunoglobulin levels as well as antigen-specific T-cell dependent humoral responses, and germinal center formation. Collectively, these studies demonstrate that immune cell numbers and functions in XLP patients can be restored by gene therapy using viral vector–mediated gene transfer [124]. As gene therapy approach usually involves autologous HSCs, there is limited risk of donor mismatch and post-transplantation-associated graft versus host disease. However, clinical success of gene therapy will require careful selection of the safest and most efficient viral vectors to ensure effective transfer of the corrected gene without inducing harmful mutagenesis.

## 10. Conclusions

It has been over three decades since the “unconventional” NK1.1^+^TCRβ^+^ T cells were first described, but it is only in the last 15 years that we have really begun to gain deeper insights into the complex network of transcription factors and signaling molecules that modulate iNKT cell development and function. In this review article we have summarized the key findings that enhance our understanding of how the SFRs, SAP, and Fyn function to regulate iNKT cell ontogeny, fate decisions, and antitumor responses. It is important to note here that the role of the SLAM-SAP-Fyn signaling axis in iNKT cell functions is not limited to their cytokine production and/or cytotoxicity. SAP expression in iNKT cells is also required for cognate help to facilitate B-cell responses [113]. Interestingly, recent studies demonstrate that SAP is also required for the development and functions of the less understood type II NKT cells [125,126]. These findings raise some intriguing questions. Given their diversity, do all the type II subsets rely on SAP for their development? If so, does the lack of type II NKT cells also contribute to the pathogenesis of XLP? Do type II NKT cells also utilize the same mechanisms to kill target cells, as do their type I counterparts? Does SAP associate with Fyn to regulate type II NKT cell development and/or their function? Do SFRs contribute positively or negatively to type II NKT cell development? These studies are significant as they are likely to assist our understanding of whether and how the various NKT cell subsets co-operate to mediate their functions, not only in XLP or cancer patients but also in other disease settings.

The severe phenotype of the *Sap*^−/−^ mice as compared to SFR-KO or Fyn-deficient mice indicates involvement of other receptors. Although recent studies have elegantly unraveled the mechanisms by which the SFRs contribute to iNKT cell development [86,93], it still remains unclear whether specific SFRs are required during the distinct stages of development. If all the SFRs are not required equally or during all the stages of iNKT cell ontogeny and maturation, what factors govern their expression and functions at the different stages? Do distinct SFRs drive lineage expansion of the specific iNKT cell subsets? Do SFRs regulate specific transcription factors that control iNKT cell fate decisions? While it has been known for years that PLZF is critical for proper iNKT cell development and acquisition of the memory phenotype, only recently have studies provided evidence that directly connect the SFR signals with PLZF expression [86,93]. Additionally, it is not known which transcription factors regulate the SFR, SAP and Fyn expression in DP cells. Studies have shown that cells lacking the transcription factors c-Myb [127] or HEB [128] exhibit reduced expression of SLAM, Ly108, SAP, and Fyn, suggesting that these molecules are also subject to transcriptional regulation, most likely by the same factors that dictate early stages of lineage commitment. With regards to function, recent studies implicate Ly9 as a negative regulator of iNKT cell cytokine production [99]; however, additional studies are required to identify which SFRs are activating receptors for iNKT cell cytokine production. Finally, will the results of these studies translate to humans? Given the momentum of the last decade, it is very likely that the future studies will provide answers to these questions and lend deeper understanding into the signaling mechanisms that regulate iNKT cell development and function. These studies will aid us in designing effective strategies to manipulate iNKT cell numbers and/or function and to treat clinical conditions, where iNKT cells are known to play either protective or pathogenic roles.
